# The Potential Links between lncRNAs and Drug Tolerance in Lung Adenocarcinoma

**DOI:** 10.3390/genes15070906

**Published:** 2024-07-11

**Authors:** William J. H. Davis, Catherine J. Drummond, Sarah Diermeier, Glen Reid

**Affiliations:** 1 Department of Pathology, Dunedin School of Medicine, University of Otago, P.O. Box 56, Dunedin 9054, New Zealand; davwi924@student.otago.ac.nz (W.J.H.D.); cath.drummond@otago.ac.nz (C.J.D.); 2 Maurice Wilkins Centre for Molecular Biodiscovery, The University of Auckland, Private Bag, Auckland 1023, New Zealand; 3 Department of Biochemistry, University of Otago, P.O. Box 56, Dunedin 9054, New Zealand; sarah.diermeier@otago.ac.nz; 4 Amaroq Therapeutics, Auckland 1010, New Zealand

**Keywords:** lncRNA, drug tolerance, targeted therapy, lung adenocarcinoma, acquired drug resistance

## Abstract

Lung cancer patients treated with targeted therapies frequently respond well but invariably relapse due to the development of drug resistance. Drug resistance is in part mediated by a subset of cancer cells termed “drug-tolerant persisters” (DTPs), which enter a dormant, slow-cycling state that enables them to survive drug exposure. DTPs also exhibit stem cell-like characteristics, broad epigenetic reprogramming, altered metabolism, and a mutagenic phenotype mediated by adaptive mutability. While several studies have characterised the transcriptional changes that lead to the altered phenotypes exhibited in DTPs, these studies have focused predominantly on protein coding changes. As long non-coding RNAs (lncRNAs) are also implicated in the phenotypes altered in DTPs, it is likely that they play a role in the biology of drug tolerance. In this review, we outline how lncRNAs may contribute to the key characteristics of DTPs, their potential roles in tolerance to targeted therapies, and the emergence of genetic resistance in lung adenocarcinoma.

## 1. Introduction

**Figure 1:**
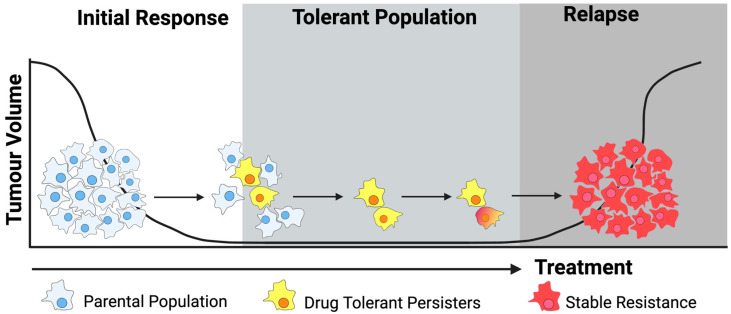
Drug-tolerant persisters lead to drug resistance and relapse. Adaptive cell responses lead to resistance to targeted therapies via the emergence of drug-tolerant persisters (DTPs). DTPs enter a reversible dormant state that allows them to survive treatment [3] and become a reservoir for the development of resistance-conferring mutation, which leads to stable genetic resistance [7].

Patients diagnosed with oncogene-driven lung adenocarcinoma (LUAD) frequently respond well to targeted therapies but invariably relapse due to the development of drug resistance [1]. Although Darwinian inheritance and the selection of pre-existing resistance-conferring mutations are widely accepted as causes of resistance, the idea that non-genetic changes may lead to resistance has gained traction in recent years [2]. Recent studies have shown that drug resistance emerges in part from a subset of cancer cells termed “drug-tolerant persisters” (DTPs), which enter a dormant, slow-cycling state that enables them to survive drug treatment and persist in patients [3] (Figure 1). The term drug-tolerant persister is made of two loosely defined terms with origins in the field of microbiology: tolerance, which refers to a non-genetic state of dormancy; and persistence, which refers to the ability of DTPs to survive despite ongoing treatment [3,4]. Drug tolerance is a phenomenon distinct from drug resistance, being entirely reversible upon drug withdrawal. DTPs act as a pool of surviving cells [3,5] from which a stable (genetic) resistant tumour cell population can arise, leading to relapse [6,7].

**Figure 2:**
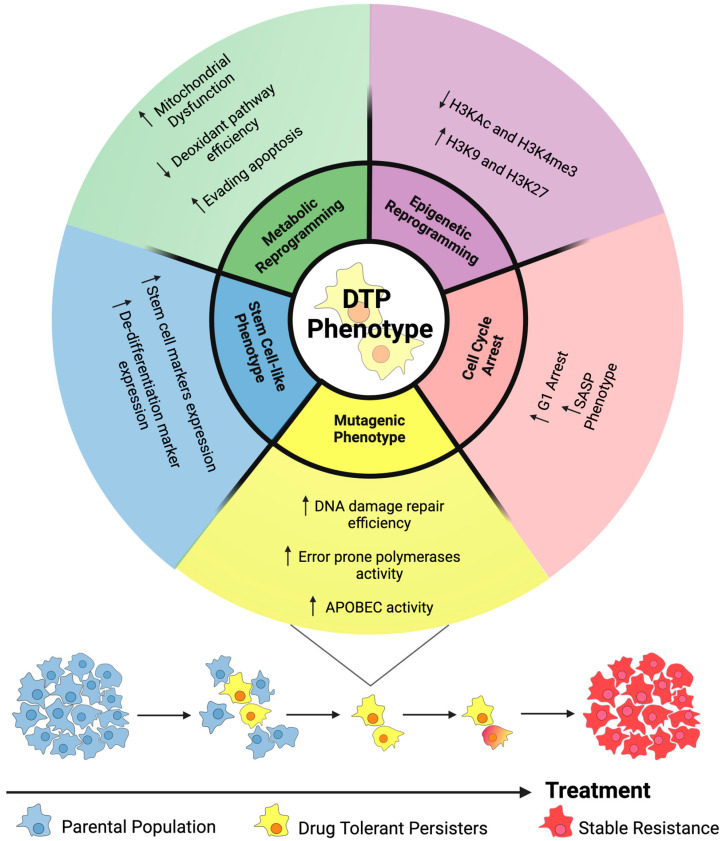
Key features of drug tolerance. Drug tolerant persisters (DTPs) exhibit several characteristic features including metabolic and epigenetic reprogramming, cell cycle arrest, and stem-cell-like and mutagenic phenotypes [13]. Arrows represent up or downregulation of the pathways indicated.

Increasing evidence suggests that the DTP phenotype is not limited to a specific cancer type, but is a common response of cancer cells to treatment [3,6,8,9,10]. Following early studies in melanoma [8] and LUAD [3], DTPs have subsequently been identified in a number of tumour types including colorectal [6], breast [9], and glioblastomas [10]. Based on the characteristics of DTPs observed in these different tumour types, several key features of drug tolerance can be proposed, such as broad epigenetic reprogramming, slowed proliferation, treatment-induced cellular senescence-associated secretory phenotype (SASP), stem cell-like characteristics, metabolic reprogramming and a mutagenic phenotype mediated by adaptive mutability (Figure 2) [2,3,5,11]. These characteristics are largely attributed to extensive transcriptional reprogramming upon drug treatment, which has also been demonstrated to lead to the altered phenotype exhibited by DTPs [12]. Despite this, the possible role of long non-coding RNAs (lncRNAs) in mediating these phenotypes in DTPs has been overlooked, leaving a significant gap in our understanding of this population.

## 2. Long Noncoding RNAs

LncRNAs are arbitrarily defined as non-coding RNA transcripts longer than 200 nucleotides in length [14]. This highly heterogenous class of RNA is generally, but not always, transcribed by RNA polymerase II [15] and includes sub-classes such as intronic, antisense, intergenic, and circular lncRNAs [16]. Once dismissed as simply transcriptional noise [17]—the so-called “dark matter” of the genome—lncRNAs have emerged as important players in the physiology and pathology of the cell [14]. LncRNAs participate in complex regulatory networks and can act in close spatial proximity around their site of transcription (in cis), and/or at distant loci (in trans). This allows lncRNAs to regulate processes in the nucleus or cytoplasm [15,16]. Their functions are often highly context-dependent and tissue-specific [15,18], and through multiple mechanisms, they can regulate a range of cellular components including proteins, mRNAs, other non-coding RNAs, and epigenetic complexes [14,15,16,18]. Given the diverse nature of lncRNA regulation, our understanding of their molecular mechanisms and how they contribute to disease pathologies [15], including cancers [18], remains in its infancy [14]. The involvement of lncRNAs in tumour biology is broad, with evidence already suggesting that lncRNAs play key roles in drug resistance and relapse [19].

## 3. Drug Tolerance

While the role of lncRNAs in drug tolerance is still largely unknown, recent transcriptomic analyses implicate several lncRNAs as potential key players in this phenomenon [12]. Consistent with this, a number of lncRNAs have known roles in the main features of drug tolerance, including in stem cell biology [20], cell cycle regulation [21,22], epigenetic reprogramming [15,23], DNA repair [24] and mutagenesis [25]. In this review, we describe how lncRNAs contribute to the key characteristics of drug tolerance to outline their potential importance in tolerance to targeted therapies and the emergence of genetic resistance in LUAD.

### 3.1. Epigenetic Reprogramming

DTPs exhibit a widespread chromatin-mediated repressive state, with a global decrease of histone H3 lysine acetylation (H3KAc) and tri-methylation at lysine 4 (H3K4me3), and an increase in methylation of lysine 9 (H3K9) and lysine 27 (H3K27), which alters their transcriptomic landscape [26,27] This repressive chromatin state is likely to lead to the subsequent downregulation of lncRNAs that could impact the response of the cell to drug treatment. For example, it has been widely reported that promotor hypermethylation of *maternally expressed 3* (*MEG3*) leads to its downregulation [28]. Considered to function as a tumour suppressor, *MEG3* is downregulated in LUAD [29]. It has been shown to positively regulate transcription of *TP53* mRNA while having repressive effects on MDM2, which negatively regulates p53 protein, thus enhancing the p53 responses to cell stress [30,31]. *MEG3* is thought to function using unique tertiary structural motifs that form pseudoknot structures called “kissing loops” [32]. Due to the generally repressive chromatin state of DTPs, the downregulation of lncRNAs such as *MEG3* may promote drug tolerance and ultimately drug resistance.

Given that lncRNAs are thought to extensively regulate chromatin [15,23], lncRNAs may contribute to the epigenetic landscape present in LUAD DTPs. A diverse array of RNA molecules, including lncRNAs, are chromatin enriched and able to regulate chromatin compaction and thus gene expression [15,33,34]. As an RNA molecule, lncRNAs have the inherent ability to counter positively charged histone tails, and as such their association with chromatin could lead to chromatin de-compaction [15,35]. Additionally, lncRNAs are known to interact with chromatin through several other modes, including regulating histone compaction, forming DNA-RNA hybrids and R-loops, and regulating and guiding protein interactions with DNA [15,36]. An example of this is the lncRNA *just proximal to Xist* (*Jpx*), which regulates chromatin looping by displacing the CCCTC-binding factor (CTCF) from regions of the genome associated with early cell differentiation and development [37].

DNA methylation regulates gene expression via methyl group modification of cytosine residues [38] and is largely regulated by DNA methyltransferases, specifically DNMT1, DNMT3a, and DNMT3b in mammals [39]. To study interactions between lncRNAs and proteins such as epigenetic modifiers, RNA-precipitation methods have been developed. Methods such as RNA immunoprecipitation sequencing (RIP-seq) and cross-linking and immunoprecipitation (CLIP) have emerged as powerful tools to study interactions with DNMT enzymes and the lncRNAs that regulate them [38,40]. For example, RIP-seq was used to identify lncRNAs that associated with DNMT enzymes in colorectal cancer [39]. This study identified the lncRNA DACOR1, which directly interacted with DNMT1. Interestingly, the enrichment of DACOR1 resulted in the downregulation of negative regulators of the S-adenosyl methionine pathway, a key methyl donor for methylation [39]. Similar precipitation methods have also been used to study the interaction between histone acetylation and lncRNAs. For example, photoactivatable ribonucleoside–enhanced cross-linking and immunoprecipitation (PAR-CLIP) was used to demonstrate interactions between CREB-binding protein (CBP) enhancer RNAs, including lncRNAs [41]. These RNAs bind directly to the histone acetyltransferase domain of CBP and promote histone acetylation [41].

Polycomb repressive complexes (PRCs) mediate the addition of generally repressive methylation marks to regulate gene transcription [42], including those known to be dysregulated in LUAD DTPs [3,26,27]. A very large number of lncRNAs are associated with PRCs in human tissues [43], and while PRCs are highly promiscuous RNA binding partners [44], evidence suggests that a number of these lncRNAs may contribute to chromatin changes in DTPs. Knockdown of several lncRNAs, including *HOX transcript antisense intergenic RNA* (*HOTAIR*), resulted in derepression of PRC2 target genes supporting a role for these lncRNAs in contributing to PRC2 activity [14,43]. Furthermore, recent studies have indicated a necessity for RNA/PRC2 interactions in PRC2 chromatin occupancy and function [45]. While there remains much debate in the field [14], this evidence suggests that at least some lncRNAs are involved in regulating PRCs, which may contribute to the widespread dysregulation of the epigenetic landscape seen in drug tolerance.

**Figure 3:**
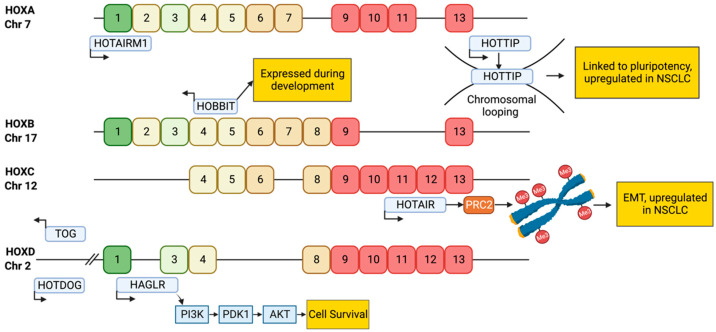
Non-Coding RNA Regulation of *HOX* Loci. *HOX* loci are thought to be extensively regulated by lncRNAs, including the lncRNAs HOTAIR and HOTTIP [47]. *HOX* genes play an important role in development and cell plasticity [48], which are phenotypes widely seen in drug tolerance in LUAD, and thus may be important players in this response. *HOX* genes are arranged in 4 clusters in humans, with each cluster containing several *HOX* genes (numbers shown). *HOX* genes are expressed spatially during embryogenesis, with green and yellow colours representing *HOX* genes expressed anteriorly and centrally, and red representing *HOX* genes expressed posteriorly in humans.

*HOTAIR* is a 2158 nucleotide trans-acting lncRNA involved in the regulation of homeobox (*HOX*) genes [46]. *HOTAIR* is a transcribed antisense of the *HOXC* gene and is one of the most studied lncRNAs [23,46]. *HOX* genes are found in highly evolutionarily conserved genetic regions and play an essential role as transcription factors during development as well as carcinogenesis in a number of cancers including LUAD [47,48,49]. Both coding and non-coding *HOX* genes extensively regulate stem cell and developmental programs involved in differentiation, de-differentiation, and cell identity [47,48,49,50,51] and could have important implications in DTPs (Figure 3) which exhibit a de-differentiated and stem cell-like phenotype (see below) [3,5]. The regulation of *HOX* genes occurs via tightly linked chromatin/ncRNA interactions [47], with intergenic regions flanking *HOX* genes extensively transcribed into lncRNAs that regulate *HOX* gene transcription [49] predominantly by recruiting chromatin-modifying proteins [46,48].

As a trans-acting lncRNA, *HOTAIR* is thought to regulate PRC2-mediated methylation both within the *HOX* gene and throughout the genome [52,53,54]. Interaction between HOTAIR and PRC2 is reported to modulate the enzymatic activity of PRC2 increasing the incidence of PRC2-mediated methylation [47]. *HOTAIR* primarily regulates transcription of the *HOX* gene cluster by directly recruiting PRC2 to methylate histones in the promoter of *HOXD* and other developmental targets throughout the genome [23,46]. When overexpressed in cancer this results in increasing de-differentiation [52,53]. In addition, *HOTAIR* is able to bind to the LSD1 complex, a lysine demethylase that promotes repressive demethylation of H3K4 [55] resulting in the induction of epithelial to mesenchymal transition [51,55]. High expression of *HOTAIR* is implicated in progression and is indicative of poor prognosis in LUAD [56]. Its expression is also linked to resistance to both chemotherapies and targeted therapies [49,57], which further indicates a role for *HOTAIR* in the emergence of DTPs.

Another well-characterised lncRNA emerging from the *HOX* locus is *HOXA Distal Transcript Antisense RNA* (*HOTTIP*) [58], a 3764 nucleotide long lncRNA [58] transcribed from the 5′ end of the HOXA gene. Chromosomal looping facilitates interactions between the *HOTTIP* lncRNA and target genes [58], after which *HOTTIP* recruits the adapter protein WD repeat-containing protein 5 (WDR5), which enables the lysine methyltransferase KMT2A to add methylation marks to regions throughout target genes including HOXA [58]. *HOTTIP* is actively expressed throughout development [58] and like *HOTAIR*, *HOTTIP* upregulation is common in patients with LUAD. While overexpression is correlated with poor overall survival [59,60], functional studies on *HOTTIP* in LUAD remain limited.

### 3.2. Slow Cycling Phenotype

The slow-cycling phenotype exhibited by LUAD DTPs upon treatment with targeted therapy [3,5,61] mimics the previously described persister populations in bacteria [62,63]. Most DTPs exist in a state of quiescence by failing to proceed past the G1 phase of the cell cycle [3,5,61] although recent evidence suggests that a small population continues to cycle despite MAPK inhibition [5]. Importantly, cycling and non-cycling DTPs are genetically identical and exhibit reversible phenotype switching, and reacquire drug sensitivity following withdrawal [5]. Although studies into cell cycle-related lncRNAs have yet to be performed specifically in DTPs, our understanding of lncRNAs that regulate the cell cycle, particularly in a cancer cell context [21,22], points towards their involvement in the slow cycling phenotype that is evident in DTPs.

Progression through cell cycle checkpoints is governed by cyclins and cyclin-dependant kinases (CDKs), which themselves are regulated by an ever-increasing number of lncRNAs [21,22]. For example, the levels of *Metastasis-Associated Lung Adenocarcinoma Transcript 1* (*MALAT1)* are known to fluctuate between cell cycle phases [64]. *MALAT1* is required for progression through the cell cycle, and its depletion using antisense oligonucleotides (ASOs) resulted in an increased fraction of cells arresting at the G1 phase and becoming quiescent [64], an observation mirrored in DTPs [5,61]. Mechanistically, it has been proposed that *MALAT1* suppresses the expression of cyclin A2 and CDC25A, which are both required to transition from the G1 phase into the S phase [22,64]. In DTPs surviving erlotinib treatment, single-cell RNA sequencing (scRNAseq) analysis found significantly higher levels of *MALAT1* transcripts [12]. Although no further investigation was carried out, this finding suggests that *MALAT1* may play a role in drug tolerance via its involvement in cyclin regulation. Whether upregulation of MALAT1 is causative rather than consequential has yet to be addressed.

Another CDK regulating lncRNA is *p53-induced noncoding transcript* (*lincPINT*), [65] which is ubiquitously expressed in human tissues and has been shown to have tumour suppressive qualities in cancer [65,66]. Interestingly, *lincPINT* expression is downregulated significantly in a variety of cancers including LUAD [67]. *LincPINT*, like many lncRNAs, has diverse functions but appears to primarily exist as an epigenetic modifier. By interacting with EZH2 and other polycomb-associated protein complexes it silences genes, including cell cycle genes such as CDK1 [66], which regulates the G2/M phase transition [68]. *Taurine-upregulated gene 1* (*TUG1)* is also able to modulate progression through the cell cycle via interacting with PRC2 in LUAD [69,70,71]. Recent reports suggest that this association modulates the expression of the *HOX* gene *HOXB7*, which activates the AKT and MAPK pathways to regulate progression through the cell cycle [72]. Interestingly, repressed proliferation due to *TUG1* knockout results in an accumulation of cells at G1/S similar to that seen in DTPs [71].

### 3.3. Regulation of a SASP-like Phenotype

The state of dormancy adopted by LUAD DTPs in response to targeted therapy shares many of the features of treatment-induced cellular senescence and the senescence-associated secretory phenotype (SASP) [73,74,75]. This includes upregulating and secreting many SASP factors [73,74]. Staining for senescence-associated β-galactosidase, demonstrated that LUAD DTPs do indeed appear to be in a state of senescence following the addition of targeted therapies [73,74]. Following treatment with EGFR or MEK inhibitors, LUAD cells secrete SASP-associated cytokines and chemokines including IL-6, IGFBP, and MMP [73,74] and resulting DTPs exhibit elevated levels of the senescence marker p27^Kip^ [74]. Put together, it is reasonable to conclude that the SASP may be important for the development of DTPs, and would support the notion of multiple DTP populations supporting one another through tolerance by regulating cell cycle factors, as recently reported [5].

The lncRNA variously referred to as microRNA-31 host gene (*MIR31HG*), *LOC554202,* or *long noncoding HIF-1α co-activating RNA* (*LncHIFCAR*) is an essential regulator of both the expression and secretion of IL-1α and other essential SASP components by repressing p16 expression [76,77]. It was recently identified that oncogene activation drives increased expression of *MIR31HG* via an enhancer element upstream of its promoter, as well as translocation of *MIR31HG* from the cytoplasm into the nucleus. Following translocation, *MIR31HG* represses p16/CDKN2A expression by recruiting PRC2 and adding repressive H3K27me3 methylation to the p16/CDKN2A locus which prevents cell cycling [76]. *MIR31HG* also promotes RSK-mediated phosphorylation of YBX1, which in turn increases IL-1α translation further inducing SASP [77]. In addition, mTOR signalling is upregulated in DTPs [78] (see below) and is known to control the expression of IL-1α and other SASP factors at the post-transcriptional level [79]. It is noteworthy that inhibition of mTOR using rapamycin was able to reduce SASP signalling in LUAD DTPs while also reducing the number of DTPs emerging following treatment. This suggests there may be some clinical value to inhibiting SASP in DTPs [73].

Another lncRNA that regulates SASP is *ANRIL* (*antisense non-coding RNA in the INK4 locus*) [80,81]. During oncogene-induced senescence, *ANRIL* induces SASP by binding to SUZ12, a component of the PRC2 complex [80,82,83], recruiting PRC2 and mediating repression of p15 and p16, thus inhibiting the cell cycle by regulating CDKs [80,81,83,84]. As a result, knockdown of *ANRIL* decreases proliferation as p15 and p16 expression are increased, inducing SASP [81,84]. Regulation of *ANRIL* activity is complex and determined by methylation, transcription factor binding activity, splicing, and miRNAs [85]. It should be noted that *ANRIL* is negatively regulated by RAS signalling [80,84] as well as by DNA damage [86], potentially linking *ANRIL* to the SASP-like DTP phenotype.

### 3.4. Stem and Developmental Cell-like Features in DTPs

One of the most frequently reported features of drug tolerance is the emergence of a stem cell-like phenotype following drug treatment [2,5,12]. It is now well established that DTPs adopt a stem cell-like phenotype, exhibiting high levels of stem cell markers including the Yamanaka factors OCT3/4, SOX2, and NANOG, as well as CD133 and ALDH [10,73,87]. Interestingly, DTPs also appear to shift phenotypically towards a progenitor cell type, with melanoma DTPs adopting a neural crest-like phenotype and LUAD DTPs adopting a phenotype reminiscent of the multipotent alveolar type 2 (AT2) lung progenitor cells [11,12].

SOX2, OCT3/4, and NANOG are central to pluripotency during stem cell development [88]. These transcription factors have been detected in several DTP populations in various cancer types [8,10,73,89] and lncRNAs are involved in their regulation [90,91]. The protein-coding *SOX2* gene is located on chromosome 3q26.3 within an extensive intronic region of the larger non-coding gene *SOX2OT* (*SOX2 overlapping transcript*) [92]. *SOX2OT* is a large lncRNA transcribed sense along with SOX2 and co-expressed with the other core pluripotency factors [92,93,94]. In lung cancer, *SOX2OT* is often overexpressed, particularly in squamous cell carcinomas [92,95]. Functionally, evidence suggests that *SOX2OT* is able to regulate SOX2 in cis, and other pluripotency factors, including OCT3/4, in trans [92].

*LncTCF7* is another lncRNA that regulates pluripotency factors [96]. *LncTCF7* was found to be highly abundant in cancer stem cell (CSC) fractions derived both from cell lines and from patient samples enriched for the CSC markers CD13 and CD133 [96]. *LncTCF7* acts to induce the transcription of TCF7, which is known to regulate signalling cascades induced in CSCs, including Wnt signalling [96]. Knockdown of *lncTCF7* using shRNAs resulted in significantly reduced expression of *OCT3/4*, *NANOG,* and *SOX2*, potentially by regulating TCF7 in cis, and significantly hampered the emergence of a CSC fraction [96]. The *colon cancer-associated transcript 1* (*CCAT1*) [97], sometimes referred to as *CARLo-5*, also interacts with the SOX2 protein, as well as the p63 protein. This complex activates MAPK and PI3K/AKT signalling, and offers a potential bypass mechanism for oncogene pathway reactivation or bypass following inhibition via targeted therapies [97]. Additionally, *CCAT1* is part of an inhibitory feedback loop with p63, which could potentially limit apoptotic entry [97,98]. Together, these observations indicate that lncRNAs can play a significant role in regulating stemness phenotypes, such as is found in the DTP phenotype.

**Figure 4:**
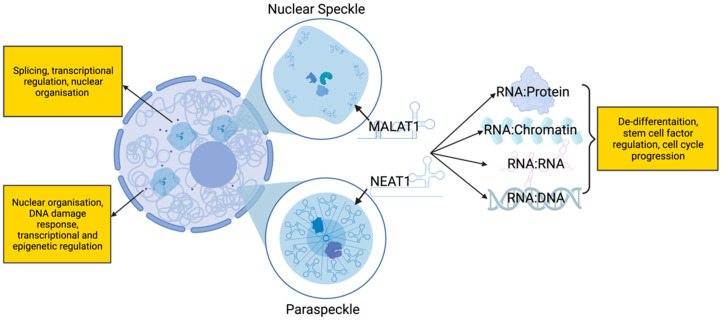
Potential roles of MALAT1 and NEAT1 in the DTP phenotype. Both MALAT1 and NEAT1 are architectural lncRNAs involved in nuclear organisation and regulation [103,104]. They also regulate protein, chromatin, RNA, and DNA independently of their architectural roles. These functions include the regulation of several characteristics of DTPs, including but not limited to chromatin remodelling, stem cell factor regulation, de-differentiation, and DNA damage responses.

Interestingly, *MALAT1* and *Nuclear Paraspeckle Assembly Transcript 1* (*NEAT1*) are also transcriptionally regulated by OCT3/4, with evidence that OCT3/4 binds to the promoter of *MALAT1* while acting as an enhancer for *NEAT1*, thereby increasing their expression in lung cancer [99]. *MALAT1* and *NEAT1* are highly conserved lncRNAs that are abundantly expressed in multiple tissues and are tightly linked to the regulation of stem cell factors and processes involved in cell plasticity and tumour cell de-differentiation (Figure 4) [99,100,101,102]. A recent single-cell RNA-sequencing analysis investigated temporal changes in gene expression in DTPs in LUAD [12]. While the study focused on changes in protein-coding RNA expression, it was noted that *MALAT1* and *NEAT1* were among some of the most highly differentially expressed transcripts in DTPs [12]. Although no follow-up studies on the possible contribution of these transcripts in the biology of drug tolerance have been performed, several known functions of *MALAT1* and *NEAT1* suggest that they play a role in drug tolerance.

*MALAT1* encodes a ~8 kb transcript, referred to as *MALAT1*, from which a short 61 nucleotide cytoplasmic RNA known as *MALAT1*-associated small cytoplasmic RNA (*mascRNA*) is cleaved from the 3′ end [105,106]. *NEAT1* forms two distinct isoforms: a long, 22.7 kb isoform, designated *NEAT1_2*, and a short 3.7 kb isoform, designated *NEAT1_1* [107]. Both *MALAT1* and *NEAT1* have diverse roles in regulating many processes that could have important implications for drug tolerance. Of particular interest in this context are the roles of *MALAT1* and *NEAT1* in regulating stem cell factors, in particular, *OCT3/4* and *SOX2*, in LUAD [99,102] and other cancer types [100,101] thus having the potential to be important factors in drug tolerance. *MALAT1* expression also appears to be tightly linked to increased de-differentiation and increased metastatic phenotypes in lung cancers [108,109] and, furthermore, *NEAT1* is found in CSC fractions of LUAD cells [102].

As well as regulating processes as an independent lncRNA molecule, the long *NEAT1* isoform, *NEAT1_2* is also an indispensable structural scaffold for nuclear paraspeckles [110,111]. Paraspeckles are a liquid-liquid phase separated, membrane-less nuclear body that is constructed upon *NEAT1_2* transcription [111,112]. An increasing abundance of *NEAT1* directly contributes to the assembly of more paraspeckles [111,112], thus sequestering essential paraspeckle proteins from other activities in the cell [25]. Interestingly, many essential paraspeckle proteins, such as NONO and FUS, also have important roles in DNA damage repair [25,113,114], and therefore could be implicated in adaptive mutability in drug tolerance (discussed below).

#### Developmental Lung Genes Co-Ordinated by lncRNAs

One outcome of de-differentiation and increasing plasticity is the adoption of a tissue-specific progenitor-like cell type. This phenomenon was first documented in melanoma DTPs in response to treatment with targeted therapies [89]. In LUAD, scRNAseq analysis of DTPs in vitro [12] and in residual disease in patients following treatment with targeted therapies [11], determined that surviving dormant residual cells increase their plasticity and stem-cell markers and adopt a lung progenitor-like state. These cells express markers of AT2 cells [11] which in addition to being a mature cell type responsible for producing surfactants in the alveolus, also act as stem-like lung progenitor cells [11,115]. Importantly, lncRNAs are highly involved in differentiation and development and these lncRNAs have been linked to cancers [20]. One of the first factors vital for lung development, the transcription factor NKX2-1 [116], remains high in AT2 cells [115] and is enriched in lung DTPs [11]. NKX2-1 is regulated by its antisense transcript *NKX2-1-AS1* [117,118,119], which has been found to be highly expressed in LUAD [117], gastric [118], and prostate cancers and is linked to the development of a plastic neuroendocrine phenotype [119]. Surfactant proteins, such as SFTPB, SFTPC, and SFTPD, are additional markers of AT2 cells [115] that are enriched in drug tolerance in LUAD [11]. Surfactant homeostasis is thought to be regulated by *Surfactant Associated 1* (*SFTA1P*) [117], suggesting a role for this lncRNA in regulating the emergence of DTPs with a stem-cell-like phenotype.

### 3.5. Metabolic Reprogramming

Reprogramming a tumour’s metabolic landscape is a hallmark of cancer [120], and DTPs are known to exhibit extensively altered metabolism. This contributes to their insensitivity to targeted therapy, and to their vulnerability to drugs that interrupt the resulting precarious metabolic state [121]. Mitochondrial metabolic dysfunction is an extremely common feature of human cancers, including lung cancers [122,123].

The Warburg effect, a metabolic shift from oxidative phosphorylation (OXPHOS) toward glycolysis, is one of the most predominant metabolic changes observed in cancer cells [123]. In contrast, DTPs tend to move away from relying on glycolysis and instead exhibit increased OXPHOS-dependent metabolism [124]. Supporting this theory, the inhibition of OXPHOS was able to partially re-sensitise DTPs to MAPK inhibition, decreasing the DTP fraction of surviving cells [125]. This suggests that this switch may also contribute to tolerance rather than simply being an outcome of existing in a dormant state. Many lncRNAs are linked to the promotion of the Warburg effect [126,127] and thus may be downregulated in DTPs in response to treatment. Such lncRNAs include *lincRNA-p21*, which promotes glycolysis [128], and *NEAT1*, which acts as a scaffold for PGK1, PGAM1, and ENO1, thus promoting the transition toward glycolysis in breast cancer [129]. Similarly, the lncRNA *glycoLINC* also acts to scaffold PGK1/PGAM1/ENO1 during the Warburg effect [130]. Upregulation of *glycoLINC* dramatically increases ATP production during the Warburg effect by increasing the flux through glycolysis [130]. In contrast, several lncRNAs promote transition toward OXPHOS and therefore may be upregulated in DTPs. For example, *isocitrate dehydrogenase 1-antisense 1* (*IDH1-AS1*) [131], which negatively regulates the Warburg effect in multiple cancer types, including colon and cervical cancers, by promoting the homodimerisation of isocitrate dehydrogenase 1, the protein produced by the *IDH1* locus [131]. This promotes OXPHOS by encouraging TCA cycle activation and inhibiting HIF1α [131]. *IDH1-AS1* regulation is part of a feedback loop with c-MYC, which is able to transcriptionally repress *IDH1-AS1*, thus resulting in the re-activation of HIF1α and promoting the Warburg effect [131].

DTPs exhibit a global reduction in the activity of redox regulators and an increase in reactive oxygen species. Whether increasing ROS confers a survival benefit to DTP is under debate, with some suggesting that this environment contributes to adaptive mutability [6,7] (see below). The finely balanced redox state of DTPs led researchers to target this vulnerability, with GPX4 inhibition showing some success [121,132]. Oxidant/antioxidant pathways and lncRNAs are thought to be tightly interlinked, although this is still an emerging field [126]. One example is *nuclear lung cancer-associated transcript 1* (*NLUCAT1*) [126,133] which is upregulated through hypoxic signalling, largely via NF-κB and NRF2, and is associated with poor prognosis in LUAD. Mechanistically *NLUCAT1* is thought to function by regulating several of the major antioxidant players, such as ALDH3A1, GPX2, GLRX, and PDK4 [133].

### 3.6. Adaptive Mutability

Resistance to targeted therapies is a frequent treatment outcome in the clinic, even in tumours that do not contain an intrinsic mechanism to bypass the therapy [1]. Recent literature has shown that cancer cells can enter a state of adaptive mutability to gain de novo mutations during treatment [7,78,134]. It is now well established that DTPs act as a reservoir of cells in which mutations can occur, likely resulting in stable resistance and tumour recurrence [3,6,61,135]. Recent evidence suggests that DTPs engage in adaptive mutability in a similar way to bacterial persisters [6,7,62,134,135]. In colorectal DTPs, DNA mismatch repair, homologous recombination, and high fidelity polymerase were inhibited in response to MAPK inhibition, while error-prone polymerases were upregulated [6], consistent with previous work in which inhibition of phosphatidylinositol 3-kinase (PI3K) signals through ERK suppressed homologous repair in breast cancer [136]. Similar findings have now been observed in melanoma and LUAD, where DTPs showed decreased MMR and HR coupled with a down regulation in high-fidelity polymerases and elevated error-prone polymerases [7,137].

Recent evidence suggests that DTPs harness the AXL signalling pathway to progress towards a state that permits adaptive mutability [6,7,78]. AXL signalling is linked to translesion polymerase recruitment by activating RAD18 via neddylation [7]. The primary ligand for AXL activation is growth arrest-specific protein 6 (GAS6] [138], which is dramatically enriched in DTPs following treatment with targeted therapy in DTPs [7]. A major regulator of GAS6 is the antisense lncRNA *GAS6-AS1* [139]. In *cis*, *GAS6-AS1* regulates GAS6 both at the transcriptional and post-transcriptional level through the formation of RNA-RNA duplexes which are proposed to protect GAS6 transcripts from degradation by ribonucleases [140]. This increases the activation of GAS6, thus increasing the activation of AXL and its downstream pathways [140]. *GAS6-AS1 trans* regulation is more diverse, having seemingly oncogenic and tumour suppressive qualities in an array of cancers including LUAD [139], and includes regulating S-phase entry [140] as well as glucose metabolism [139,141].

Downstream of the AXL receptor is the mammalian target of rapamycin (mTOR) [138] with recent evidence suggesting both are central players in the adaptive mutability of DTPs [78]. Like AXL, mTOR is also regulated by a host of lncRNAs. For instance, the lncRNA *H19* controls the mTORC1 downstream pathway by blocking mTORC1 mediated phosphorylation of 4E-BP1 [142]. There is also evidence that well-characterised lncRNAs such as *MALAT1* and *urothelial carcinoma-associated 1* (*UCA1*) regulate processes upstream of mTOR and that this indirect regulation of mTOR could largely be behind the involvement of these lncRNAs in cancer processes [143,144].

## 4. Therapeutic Challenges and Potential

With lncRNAs emerging as essential regulators in cancer, much effort has been made to specifically target them. The main approaches have employed small-interfering RNAs (siRNAs) and antisense oligonucleotides (ASOs) [145,146]. siRNAs induce the degradation of the target RNA by recruiting the RNA-induced silencing complex (RISC), a process that takes place in the cytoplasm. In contrast, gapmer ASOs degrade target RNA through recruiting RNase H, which cleaves the double-stranded ASO-target RNA duplex. Crucially, RNaseH is also present in the nucleus where many lncRNAs localise [15,145]. ASOs have become a popular method for knocking down lncRNAs [145] including those of potential interest in drug tolerance, such as *MALAT1* [109] and *NEAT1* [147]. Another approach to target lncRNAs is the use of CRISPR-Cas systems. These are able to inactivate genes on the DNA level through deletions as well as activate (CRISPRa) [148] or inhibit (CRISPRi) [149] transcription, or even target mature RNAs for degradation [150]. Such systems are actively being explored in the field of lncRNAs to not only activate or inhibit activity but to precisely modulate and fine-tune the expression of lncRNAs [15,148,149,150]. Notable examples are lncRNAs *MALAT1* and *UCA1* which have successfully been the target of CRISPR-Cas-based systems [151].

While siRNA and ASO-based approaches toward targeting lncRNAs in vitro have gained momentum over other therapeutic approaches, translating such approaches into the clinic remains challenging. This is largely due to drug delivery and pharmacokinetic challenges dramatically limiting the efficacy of nucleic acid-based therapeutic approaches. Successfully delivering high concentrations to target cells, while limiting toxicity and endosomal trapping remains a significant obstacle that has yet to be overcome [146,152]. RNA base and sugar modifications, such as 2′-O-methyl and phosphorothioate modifications [146], have significantly increased stability and specificity for nucleic acid-based approaches, while also improving immunogenicity issues [146]. Antisense approaches have gained traction toward clinical translation, with an ASO complementary to the 3′ untranslated region of KRAS mRNA recently undergoing clinical trial. While this approach was promising in in vitro studies [153], translation to phase I clinical trial (NCT03101839) did not continue to phase II, possibly due to the non-selective binding of the ASO to wild-type KRAS [154]. ASOs have seen recent success outside the cancer field, with the SOD1 targeting ASO Tofersen recently gaining US FDA approval for the treatment of SOD1-positive amyotrophic lateral sclerosis (ALS). Multiple clinical trials have shown promising results, indicating that treatment with Tofersen leads to a reduction in neurofilament levels [155]. Another area of advancement is in drug administration, particularly through the use of inhalable nucleic acid-based approaches [146]. While not a new idea [156], it has seen rapid advancements due to the COVID-19 pandemic [157] and could offer benefits to patients with lung cancers in particular [158]. Systemic delivery is theoretically possible for CRISPR-Cas, but these systems can only deliver multiple RNA and protein components into cells to function, thus limiting the efficacy of the system while increasing immunogenicity issues [159]

Designing small molecules to inhibit function is becoming an increasingly popular approach to targeting lncRNAs. Small molecules have been made to target lncRNAs that contain stable 3-dimensional structures, such as the recent development of an inhibitor that binds to the 3′ triple helix structure of *MALAT1,* which is essential for the stability of the lncRNA [160,161]. Nevertheless, there are several limitations in small molecule-based approaches to targeting lncRNAs, the most glaring being a lack of knowledge of RNA structure and associated function [146]. For most lncRNAs, sufficiently accurate predictions of RNA folding and function to support small molecule design do not exist. Accurately determining 3-dimensional RNA structure is an extremely difficult task, with structure often dependent on interactions within the cell [162]. Biophysical methods are difficult to perform in a live cell context due to the dynamic structure of lncRNAs and the flexible nature of flexible RNA sugar-phosphate backbones [146,162,163]. Furthermore, when biophysical methods are appropriate, simply applying these complex and expensive techniques to the hundreds of thousands of predicted lncRNAs remains an impractical approach [15]. Even so, recent progress has been made. The in vivo click selective 2-hydroxyl acylation and profiling experiment (icSHAPE) has shown promise in measuring nucleotide flexibility in RNA on a transcriptome-wide level by tagging flexible RNA nucleobases, reverse transcribing, and sequencing these regions [164]. As computational approaches become more advanced, large language models such as those already used to “speak protein folding” may help to unravel structural folding. Given the dynamic nature of RNA folding, however, this technology may be limited [163,165]. It is also worth noting that the majority of lncRNA targeting therapeutics are restricted by the generally low sequence conservation of lncRNAs throughout the animal kingdom. While this often makes it difficult to generate pre-clinical animal models of a designed lncRNA therapeutic [14,19], these concerns can be partially mitigated with the use of human xenografts in animal preclinical models [166]. Additionally, several lncRNAs, including *MALAT1,* retain structural homology throughout the animal kingdom, particularly in the 3′ region [167], making these regions promising targets for therapeutics.

## 5. Conclusions

Despite lncRNAs being implicated in multiple processes in cancer biology, there have been few studies that investigate lncRNAs in drug tolerance. LncRNAs contribute to each of the key features of drug tolerance including epigenetic regulation, cell cycle arrest, acquisition of a stem cell state, reprogramming of metabolism, and the emergence of adaptive mutability. The relationship between lncRNAs and DTPs offers a wide range of options for identifying novel drivers of tolerance, as well as adaptive mutability and the emergence of genetic resistance. With the rise in lncRNA therapeutics and a growing understanding of drug tolerance, the role of lncRNAs is of vital importance when exploring therapeutic options for lung cancer patients.

## Data Availability

No new data were created or analyzed in this study. Data sharing is not applicable to this article.
